# Unusual presentation of histiocytosis X in the cranial vault: A rare case report

**DOI:** 10.1016/j.radcr.2024.06.002

**Published:** 2024-06-28

**Authors:** Dahmane El Hairech, Nadia El Kadmiri

**Affiliations:** a Department of Neurosurgery, Hôpital privé Clairval – Ramsay Santé, 13009 Marseille, France; b Molecular Engineering, Biotechnology and Innovation Team, Engineering, Geo-Bio-Environment, Innovation Laboratory, Polydisciplinary Faculty of Taroudant, IBN ZOHR University, Taroudannt City, Morocco

**Keywords:** Langerhans histiocytosis, Langerhans cells, Osteitis, Surgery, Case report

## Abstract

Langerhans histiocytosis or histiocytosis X is an oligo-clonal proliferation of Langerhans cells. We report the case of an 11-month-old infant who had presented with a parieto-occipital swelling since birth, which progressively increased in volume without any other presenting signs. The radiological work-up initially suspected osteitis of the vault, which was removed in its entirety. The anatomopathological study concluded that it was histiocytosis X. The extension work-up was negative. Follow-up of this patient showed no local recurrence or appearance of other localizations.

A review of the literature shows that little is known about its pathophysiology. It mainly affects children and young adults. There are several possible sites of involvement, and cranial vault involvement is a fairly frequent form among the bony sites of this pathology. The diagnosis is confirmed histologically or cytologically, and extension workup is required to confirm the diagnosis. Therapeutic management has not been standardized, and sometimes requires chemotherapy in addition to surgery. The prognosis depends on whether vital organs are affected or not.

## Introduction

Langerhans histiocytosis or histiocytosis X [1] is a rare disorder of unknown etiology. It is due to an abnormal oligo-clonal proliferation of Langerhans cells in various tissues and organs (bone, skin, lymph nodes) [2]. It can occur at any age, but is most common in children and young adults. Its incidence is estimated at 1 per 200,000 person-years. Its clinical presentation is highly diverse, depending on its extent, ranging from a simple eosinophilic granuloma to a severe multivisceral form. Three syndromes characterize variants of a single pathogenic process: eosinophilic granuloma, Hand-Schu¨ller-Christian disease and Abt-Letterer-Siwe disease [3]. Diagnosis is based on cytological examination [4]. Therapeutic management has not been standardized.

## Patient and medical observation

**Patient information:** 11-month-old infant with no particular pathological history, admitted to the emergency department for a right parieto-occipital swelling that had been present since birth. The family reported a progressive increase in volume since the age of 4 months, with skin fistulization and pus discharge.

**Clinical findings:** clinical examination revealed a slightly ulcerated swelling of the right parieto-occipital scalp, painful to palpation. Endobuccal examination did not reveal gingival ulceration.

**Fig. 1:**
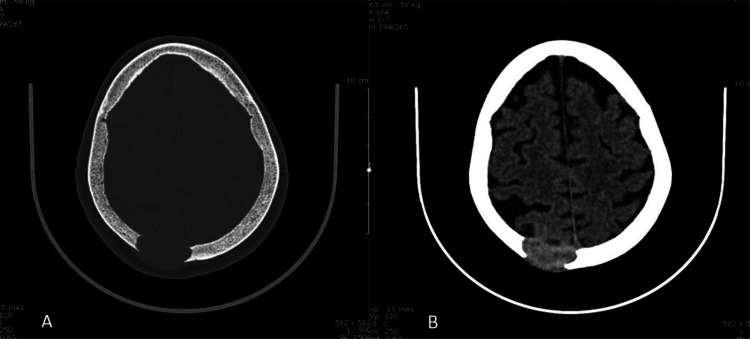
Cerebral CT axial section in bone window (A) and parenchymal window (B). Presence of a right parieto-occipital lytic bone lesion measured in its long axis 34 mm. Involvement of the periosteum and meningeum, without pial or cortical passage, is noted.

**Fig. 2:**
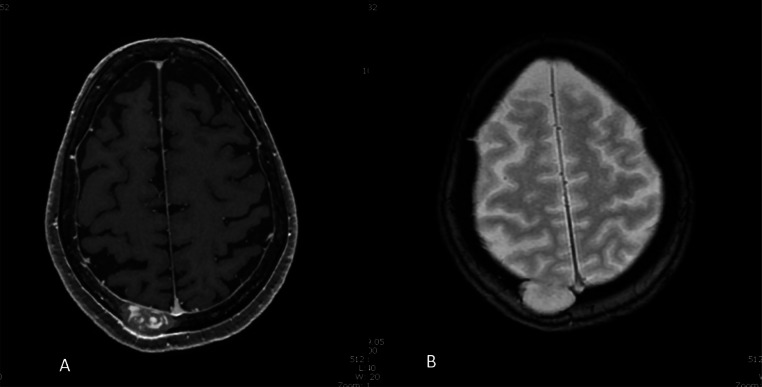
T1-weighted (A) and T2-weighted (B) brain MRI sequences: Osteolytic lesion in the right paramedian posterior parietal vault, eroding the internal and external tables, measuring 34 × 17 mm transverse diameter by 28 mm craniocaudal diameter. In depth, this lesion bulges into the epidural space, and is accompanied by slightly marked neighbouring pachymeningeal contrast. There is no oedematous reaction of the surrounding brain parenchyma.

**Diagnostic approach:** Cerebral CT scan revealed left parieto-occipital osteitis radiating to the soft tissues (Fig. 1). Biological tests were normal. Cerebral MRI showed an osteolytic lesion of the right posterior paramedian parietal vault, with millimetric, non-compressive epidural extension (Fig. 2).

**Therapeutic intervention and follow-up:** the patient underwent surgery to remove a white-grayish tumor with little hemorrhage and aspirability. Post-operative management was straightforward. Anatomopathological examination with immunohistochemical study revealed histiocytosis X. The extension work-up was negative, so follow-up without further treatment was decided.

## Discussion

Langerhans histiocytosis, also known as histiocytosis X, Letterer-Siwe disease or eosinophilic granuloma, is a rare disease defined as a proliferation of certain cells of the mononuclear system known as Langerhans cells, which contain intra-cytoplasmic Birbeck granules and display S100 protein and CD1a antigenic markers on their surface [5].

It can occur at any age, with a peak in frequency in the first three years of life. Epidemiological data on histiocytosis X are notably lacking. Its annual incidence is estimated at 1 per 200,000 children under the age of 15, with a sex ratio of 1.2 [4].

Diagnosis of certainty is confirmed by anatomopathological study, with large cells bearing the CD1a antigen on their surface [5].

Several theories have been developed concerning the pathophysiology of histiocytosis X, but these remain highly controversial, particularly the theory of neoplastic proliferation, viral etiology, somatic gene mutation or secondary to a reactive process [5,7].

It may be limited to a single location, or it may affect several different parts of the body. Various organs can be affected, essentially the bony skeleton, bone marrow, various ganglia, external auditory canals, liver, spleen, lung, central nervous system, particularly the posterior pituitary gland and digestive tract [8,9].

Cranial localization is by far the most frequent, accounting for 42% of all localizations, with temporal localization dominating in over 20% of cases [10].

Therapeutic management is based on surgery, local corticosteroid infiltration, radiotherapy and chemotherapy, although the indication for treatment remains controversial due to ignorance of the exact pathogenesis of the disease [6].

However, a few recommendations are necessary:

• In the case of single or few bone lesions, local corticosteroid infiltration or biopsy is recommended to confirm the diagnosis, or even curettage.
• In exceptional cases, radiotherapy should be used for lesions that threaten functional prognosis.
• Uni-focal forms of the disease (bone, lymph node, or skin) are generally benign and require limited therapeutic management.

## Conclusion

Langerhans histiocytosis is a rare disease, characterized by its rich clinical presentation. Its diagnostic and therapeutic protocol, rarely described in the literature, deserves to be established and validated in order to improve the prognosis of patients, particularly those with severe multi-systemic forms.

## Availability of data and materials

N/A.

## Ethics approval and consent to participate

Informed consent was obtained from the patient's parent.

## Patient consent

Written informed consent was obtained from the patient for publication of this case report and any accompanying images. A copy of the written consent is available for review by the Editor-in-Chief of this journal.

## Authors' contributions

DE, recruited the patient, designed the protocol, drafted and submitted the manuscript, NEK, revised the manuscript. All authors have approved the manuscript for sub-mission.

## References

[1] Satter EK, High WA. (2008). Langerhans cell histiocytosis: a review of the current recommendations of the Histiocyte Society. Pediatr Dermatol.

[2] Lallemant B, Fayoux P, Nelken B, Leroy X, Vaneecloo FM (2003). Du diagnostic a` la prise en charge des localisations ORL de l’histiocytose langerhansienne chez l’enfant. Ann Otolaryngol Chir Cervicofac.

[3] Dhouib M, Triki N, Karray F, Khabir A, Boudaoura T, Abdelmoula M (2006). Histiocytose langerhansienne mandibulaire. Rev Stomatol Chir Maxillofac.

[4] Bernstrand C, Björk O, Ahström L, Henter JI (1996). Intralesional steroids in Langerhans cell histiocytosis of bone. Acta Paediatr.

[5] Brichard B. (2000). Histiocytose de Langerhans : nouveautés concernant la compréhention d'une maladie énigmatique. louvain med. Vol..

[6] Gadner H, Grois N, Arico M, Broadbent V, Ceci A, Jakobson A (2001). A randomized trial of treatment for multisystem Langerhans’ cell histiocytosis. J Pediatr.

[7] Himanshu K, Sanjay B, Lily P, Apjit KC, Deepu B, Devendra KC (2004). Solitary Langerhans-cell histiocytosis of the clivus and sphenoid sinus with parasellar and petrous extensions: case report and a review of literature. Surg neurol.

[8] Luke B, Abbas E, Julie L (2006). Primary head and neck Langerhans cell histiocytosis in children. Otolaryngol–Head Neck Surg.

[9] Nezelof C, Basset F. (1998). Langerhans cell histiocytosis research. Past, present, and future. Hematol Oncol Clin North Am.

[10] Smith RJ, Evans JN. (1984). Head and neck manifestations of histiocytosis-X. Laryngoscope.

